# The Necessity of a Higher Estimation of Tooth and Mouth Hygiene

**Published:** 1895-09

**Authors:** 


### The Necessity of a Higher Estimation of Tooth and Mouth Hygiene.

Hr. P. Ritter, at the Society for Inner Medizin, pointed out how the mouth was the starting point and breeding place of germs. Now he had frequently seen workmen and workwomen lose their places on account of want of incisor teeth, or foul breath. Continuous toothache frequently led steady men to the brandy-bottle for the relief of pain. In pregnancy the necessity for attention to the mouth and teeth was especially great, as women at these times so often suffered from tooth and face ache. Probably the cause lay in excessive nausea present leading them to neglect the care of their teeth, so that the secretions became acid and favored caries. Women should be recommended to brush their teeth three times a day after the third month, using some astringent fluid and an alkaline tooth powder, and a moderately hard brush. On the basis of many years' experience, he held the following to be demanded. The appointment of experienced dentists to examine the mouths of all school children at stated intervals, the parents to be informed of the result of the examination, and left to have the treatment required carried out privately or through institutions. The appointment of dentists for the poor. Delivery of addresses in the Volksschulen on the importance of the masticatory apparatus and the toilet of the mouth. Dissemination of printed instructions on the toilet of the mouth to the whole of the poor population.

After several speakers had addressed the meeting, Professor Miller said he could completely confirm what Hr. Ritter had said as to the influence of the teeth on the whole organism. Every tooth contains a *cavum dentis*, and in this hollow lay the pulp. Through pulpitis arose toothache. The pulp then decomposed and formed an enormous number of foul-smelling masses and toxic substances that, brought under the skins of white mice, even in the smallest quantities, caused violent inflammation and suppuration. The suppuration of the pulp broke both internally and externally. Such abscess cavities never healed, but a suppuration focus permanently remained, and these could give rise to secondary processes. It was not a rarity for abscesses of the teeth to be treated by extraction. The speaker then pointed out the connection between inflammation of the tympanic cavity, and pneumonia and the oral cavity. The fact that there were healthy men with bad teeth was an exception. One frequently met children of twelve who had not a single sound tooth, and to whom every attempt to chew was painful. The roots were purulent, the gums red and inflamed, pus was constantly being formed and swallowed, and naturally the health suffered greatly.

The best means of taking care of the teeth was a brush—all other aids were accessories. The water does not pass between the teeth, but it was in these interspaces that caries began. Few people knew how to use a tooth brush properly. The brush must be small and pressed firmly into the interspaces, in order to cleanse them. Of substances to employ, many were useful, but many did harm. He could not altogether exclude salicylic acid. It was used by thousands and thousands of people, without their teeth being injured by it. Salicylic or other acids were not serviceable for every day use, but their occasional use was of advantage. Whether a remedy did harm or not could not be found out in a reagent glass, but by practice alone. He placed no value on tooth-powder; it kept the teeth white, but as it could not penetrate into the interspaces it could not prevent caries.—*Berlin Cor. Med. Press and Circular.*

